# The Effect of Precursor Concentration on the Particle Size, Crystal Size, and Optical Energy Gap of Ce_x_Sn_1−x_O_2_ Nanofabrication

**DOI:** 10.3390/nano11082143

**Published:** 2021-08-22

**Authors:** Naif Mohammed Al-Hada, Rafiziana Md. Kasmani, Hairoladenan Kasim, Abbas M. Al-Ghaili, Muneer Aziz Saleh, Essam M. Banoqitah, Abdulsalam M. Alhawsawi, Anwar Ali Baqer, Jian Liu, Shicai Xu, Qiang Li, Azlan Muhammad Noorazlan, Abdullah A. A. Ahmed, Moayad Husein Flaifel, Suriati Paiman, Nazirul Nazrin, Bandar Ali Al-Asbahi, Jihua Wang

**Affiliations:** 1 Shandong Key Laboratory of Biophysics, Institute of Biophysics, Dezhou University, Dezhou 253023, China; liujian.cn@live.com (J.L.); shicaixu@dzu.edu.cn (S.X.); qiangli_chem@hotmail.com (Q.L.); 2 School of Chemical and Energy Engineering, Universiti Teknologi Malaysia, Skudai, Johor Bahru 81310, Malaysia; rafiziana@utm.my (R.M.K.); mouneersaleh@yahoo.com (M.A.S.); 3 Department of Physics, Faculty of Applied Science, Thamar University, Dhamar 87246, Yemen; abdullah2803@gmail.com; 4 College of Computing & Informatics (CCI), Universiti Tenaga Nasional (UNITEN), Kajang 43000, Malaysia; 5 Institute of Informatics and Computing in Energy (IICE), Universiti Tenaga Nasional (UNITEN), Kajang 43000, Malaysia; 6 Department of Nuclear Engineering, Faculty of Engineering, K. A. CARE Energy Research and Innovation Center, King Abdulaziz University, P.O. Box 80204, Jeddah 21589, Saudi Arabia; ebanoqitah@kau.edu.sa (E.M.B.); amalhawsawi@kau.edu.sa (A.M.A.); 7 Center for Training & Radiation Prevention, King Abdulaziz University, P.O. Box 80204, Jeddah 21589, Saudi Arabia; 8 Department of Physics, Faculty of Science for Women, University of Baghdad, Baghdad 10071, Iraq; anwaralibaqerkram@yahoo.com; 9 Physics Department, Faculty of Science and Mathematics, University Pendidikan Sultan Idris, Tanjong Malim 35900, Malaysia; Azlanmn@fsmt.upsi.edu.my; 10 Fachbereich Physik, Center for Hybrid Nanostructures (CHyN), Universität Hamburg, 20146 Hamburg, Germany; 11 Department of Physics, College of Science, Imam Abdulrahman Bin Faisal University, P.O. Box 1982, Dammam 31441, Saudi Arabia; physci2007@gmail.com; 12 Basic and Applied Scientific Research Center, College of Science, Imam Abdulrahman Bin Faisal University, P.O. Box 1982, Dammam 31441, Saudi Arabia; 13 Department of Physics, Faculty of Science, University Putra Malaysia, Serdang 43400, Malaysia; suriati@upm.edu.my (S.P.); nazirulnazrin@ymail.com (N.N.); 14 Department of Physics & Astronomy, College of Science, King Saud University, P.O. Box 2455, Riyadh 11451, Saudi Arabia; balasbahi@ksu.edu.sa

**Keywords:** Ce_x_Sn_1−x_O_2_ nanoparticles, thermal treatment technique, polyvinylpyrrolidone, energy band gap

## Abstract

In the present work, a thermal treatment technique is applied for the synthesis of Ce_x_Sn_1−x_O_2_ nanoparticles. Using this method has developed understanding of how lower and higher precursor values affect the morphology, structure, and optical properties of Ce_x_Sn_1−x_O_2_ nanoparticles. Ce_x_Sn_1−x_O_2_ nanoparticle synthesis involves a reaction between cerium and tin sources, namely, cerium nitrate hexahydrate and tin (II) chloride dihydrate, respectively, and the capping agent, polyvinylpyrrolidone (PVP). The findings indicate that lower x values yield smaller particle size with a higher energy band gap, while higher x values yield a larger particle size with a smaller energy band gap. Thus, products with lower x values may be suitable for antibacterial activity applications as smaller particles can diffuse through the cell wall faster, while products with higher x values may be suitable for solar cell energy applications as more electrons can be generated at larger particle sizes. The synthesized samples were profiled via a number of methods, such as scanning electron microscopy (SEM), transmission electron microscopy (TEM), X-ray diffraction (XRD), and Fourier transform infrared spectroscopy (FT-IR). As revealed by the XRD pattern analysis, the Ce_x_Sn_1−x_O_2_ nanoparticles formed after calcination reflect the cubic fluorite structure and cassiterite-type tetragonal structure of Ce_x_Sn_1−x_O_2_ nanoparticles. Meanwhile, using FT-IR analysis, Ce-O and Sn-O were confirmed as the primary bonds of ready Ce_x_Sn_1−x_O_2_ nanoparticle samples, whilst TEM analysis highlighted that the average particle size was in the range 6−21 nm as the precursor concentration (Ce(NO_3_)_3_·6H_2_O) increased from 0.00 to 1.00. Moreover, the diffuse UV-visible reflectance spectra used to determine the optical band gap based on the Kubelka–Munk equation showed that an increase in x value has caused a decrease in the energy band gap and vice versa.

## 1. Introduction

Recently, nanomaterials have been the focus of extensive research studies, with their unique physiochemical properties attracting particular attention [1,2,3,4,5,6]. As a result of such studies, new systems, nanoplatforms, devices, and structures applicable to various domains have been developed [7,8,9,10]. The proliferation of related work makes nanomaterial applications demonstrating biodegradability, biocompatibility, and functionalization especially advantageous [11,12]. Empirical research has been significantly concerned with the use of cubic fluorite structures (CeO_2_) and cassiterite-type tetragonal structures of (SnO_2_) semiconductors nanomaterials [4,13,14]. Both group II and group IV elements are included in the CeO_2_ cubic fluorite structure, since it is classified as II-IV composite semiconductor [14]. There are various applications intended to exploit the singular structural features of nanomaterials on the basis of the useful chemical and physical properties [15]. It has a notable structure with a cubic fluorite structure crystalline, along with energy band gaps amounting to 3.0–3.6 eV [16]. CeO_2_ semiconductor nanostructures have wide applications, such as in photovoltaic and solar cells [17,18]. Further significant applications include diodes, clear electrodes, gas sensors, as well as antibacterial activity [19]. CeO_2_ nanostructures have been prepared in different shapes, such as nanoparticles [16], nanocrystals [20], nanoclusters [21], nanowires [22], nanotubes [23], and nanoflowers.

Similarly, the composite semiconductor (II-VI) SnO_2_-type tetragonal structure is made up of the metal Sn (II) and non-metallic elemental oxygen (VI) [4,13]. Important applications have been devised based on the remarkable properties presented by various SnO_2_ semiconductor materials [24]. Used as the archetypal tetragonal crystal structure, this material is categorized as an n-type semiconductor with 3.6 eV band gaps [25]. Research and applications have sought to exploit the characteristic properties of SnO_2_ nanomaterials rooted in their singular crystal structure and nano-sized dimensions. Thus, applications are exemplified by solar cells due to their tunable physical and chemical properties, with enhanced performance over their bulk counterparts [26,27], and optoelectronic devices [28], with applications geared towards leveraging the pellucidity occurring in the visible solar spectrum zone, as well as catalysis [29], diodes [30], gas sensors [31], and biomedical tools [32]. Furthermore, nanocrystals [33], nanoclusters [34], nanotubes [35], and nanorods [36] are among the wide range of SnO_2_ nanomaterials produced via different approaches, such as sonochemical [37], solvothermal [26], co-precipitation [38], microwave hydrothermal [39], and sol-gel treatment [40] approaches.

The complementary features are indicated by the distinguishing composition of Ce_x_Sn_1−x_O_2_ with regard to the bandgaps and sizes that grow from both oxide semiconductors. It is also probable that it has characteristics that set it apart from singular semiconductor constituents. The use of Ce_x_Sn_1−x_O_2_ for various purposes, including biocides and disinfectants is a matter that warrants attention in relation to the particular Ce_x_Sn_1−x_O_2_ nanocomposite composition. The composition displays better stability and a lengthier life than organic-based materials, not to mention the fact that it has been the focus of more extensive study with regard to biological activity [41,42]. Precipitation methods [43], co-precipitation methods [44], and the hydrothermal strategies [45] are among the techniques through which it is possible to produce Ce_x_Sn_1−x_O_2_ nanostructures. Nevertheless, due to the complexity of the process of synthesis, which involves extensive reaction times, toxic reagents, and effluent byproducts, such techniques are not highly effective for the industrial-scale production of Ce_x_Sn_1−x_O_2_ nanoparticles. Moreover, the synthesis of Ce_x_Sn_1−x_O_2_ nanoparticles at various x values has not been investigated. More specifically, an uncomplicated heat treatment technique may be adopted to avoid waste Ce_x_Sn_1−x_O_2_ nanoparticle products. The significance of this work stems from the fact that it proposes a production method compatible with industrial applications that is capable of yielding products defined by basic handling, particle size regulation (smaller and larger sizes are respectively suitable for antibacterial activity due to the smaller particle can diffuse through the cell wall faster, and energy applications due to more electrons can be generated at the bigger particle size [6]), inexpensiveness, high quality, high adaptability, a powdered form, and effective band gap. The chosen approach is also advantageous because it does not generate toxic byproducts and does not require extra chemical reagents. On the basis of this approach, this work seeks to determine how precursor values affect Ce_x_Sn_1−x_O_2_ nanoparticles.

Ce_x_Sn_1−x_O_2_ samples have been synthesized in this work utilizing a thermal-based treatment process and the effects of Ce and Sn contents on the morphological, structural, and optical properties of Ce_x_Sn_1−x_O_2_ nanoparticles have also been analyzed. The approach has involved the use of a solution with a content of metal ions and polyvinylpyrrolidone acting as precursor and capping agents, respectively. Furthermore, to obtain the desired pure nanoparticles, a calcination technique has been adopted, while various methods are applied to investigate morphology and crystallinity. The effect of x value variation is investigated as well.

## 2. Materials and Methods

### 2.1. Materials

Cerium nitrate hexahydrate with a purity of more than 99% (Ce(NO_3_)_3_·6H_2_O) and tin (II) chloride dihydrate (SnCl_2_·2H_2_O), both in concentrations of 0.00, 0.20, 0.40, 0.60, 0.80, and 1.00 mmol, were employed as the metal precursors, while polyvinylpyrrolidone and deionized water served as a capping agent, which mediated the spread of particles, and as solvent, respectively. All the chemicals were acquired from Sigma-Aldrich (US), their quality was research-grade, and none were purified further.

### 2.2. Synthesis of Samples

The preparation of the Ce_x_Sn_1−x_O_2_ nanoparticle product involved the dissolution of 4.5 g of polyvinylpyrrolidone into 100 mL of deionized water, followed by energetic stirring for 120 min at a temperature of 70 °C. The next step was the dissolution of Ce (NO_3_)_3_·6H_2_O into amounts of 0.00, 0.20, 0.40, 0.60, 0.80, and 1.00 mmol. A homogeneous solution was obtained by adding and energetically mixing SnCl_2_·2H_2_O in amounts of 1.00, 0.80, 0.60, 0.40, 0.20, and 0.00 mmol. The combined solution was placed on a petri dish and subjected to drying in an oven for one day at 80 °C. In this way, a solid was attained which was then rendered into a powdered form through crushing for half an hour in a mortar. A box furnace was subsequently employed to subject the powder to calcination at 650 °C for an hour and a half. Once the synthesized and calcined oxide nanoparticle samples were obtained, their profiling could be initiated.

## 3. Results and Discussion

### 3.1. Mechanism of the Formation of the Nanoparticle

**Figure 1:**
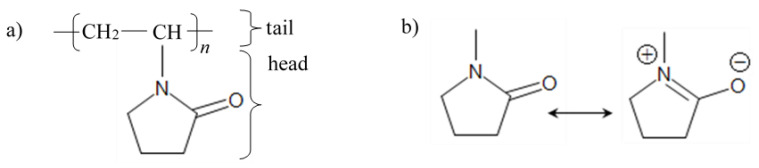
(**a**) PVP chemical structure; (**b**) formation of the resonance structure of the pyrene ring of PVP.

**Figure 2:**
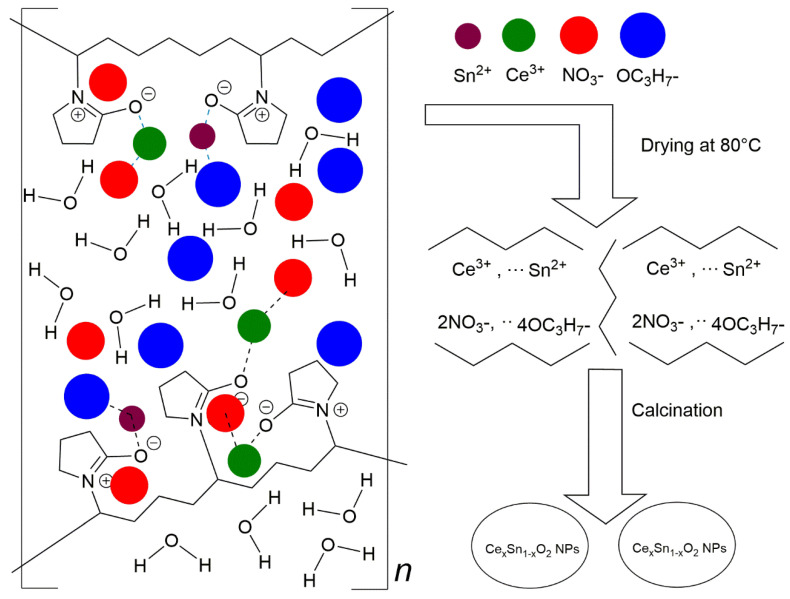
Schematic illustration of the proposed mechanism of interaction between metal ions and PVP.

Figure 1a presents the chemical structure of the amphiphilic PVP, in which the head group is the pyrrolidone part (hydrophilic) while the tail group is the polyvinyl part (hydrophobic); however, when the PVP molecules are in an aqueous solution, the structure may transform to a resonance structure as illustrated in Figure 1b [46]. Figure 2 schematically shows the process of interaction between the capping agent PVP and the metal ions. The cerium and tin are bound by strong ionic bonds between the metallic ions and the amide groups of the polymer chains. At the same time, cerium and tin ions are already bound to nitrate ions (NO_3_^−^) and the propane-2-olate ions (OC_3_H_7_^−^), respectively, via a strong ionic bond. Eventually, the metal cations are immobile in the cavities of the polymer chains, which leads to the formation of uniformly distributed metal oxides in a solid solution in the drying and calcination process [47].

PVP’s main role in the employed method is to control the growth of the particles by forming passivation layers around the metal. In addition, the functions of PVP during the preparation of Ce_x_Sn_1−x_O_2_ nanoparticles are to regulate the expansion nucleation of nanoparticles, limit the accretion of the nanoparticles, improve the degree of crystallinity of the nanoparticles, create a restricted environment around the Ce_x_Sn_1−x_O_2_ nanoparticles, and facilitate the development of nanoparticles with a homogenous dispersal of both size and form [47,48]. The drying process may decompose the PVP partly to feature shorter polymer chains [49,50]. The shortening of polymer chains causes the metallic ions to be well dispersed throughout the PVP cavities and a reduction in the number of metal ions that are being capped. The effect of PVP is not restricted only to the solution and drying steps, but also the formation of Ce_x_Sn_1−x_O_2_ nanoparticles in the calcination process through the nucleation, solid-state reaction, and the oxidization of Ce^3+^ and Sn^2+^ ions. In addition, during the calcination process, organic materials (PVP) are being eliminated, which causes steric hindrance disruption.

### 3.2. TEM Analysis

**Figure 3:**
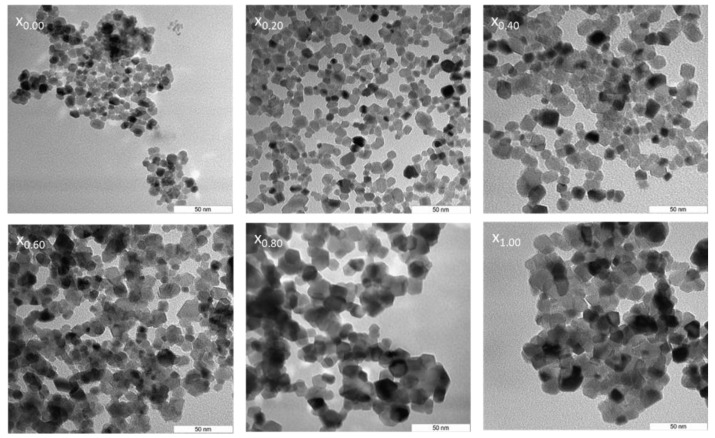
TEM images of (x_0.00_) Ce_0.00_Sn_1.00_O_2_, (x_0.20_) Ce_0.20_Sn_0.80_O_2_, (x_0.40_) Ce_0.40_Sn_0.60_O_2_, (x_0.06_) Ce_0.60_Sn_0.40_O_2_, (x_0.08_) Ce_0.80_Sn_0.20_O_2_ and (x_1.00_) Ce_1.00_Sn_0.00_O_2_) nanoparticles calcined at 650 °C.

**Figure 4:**
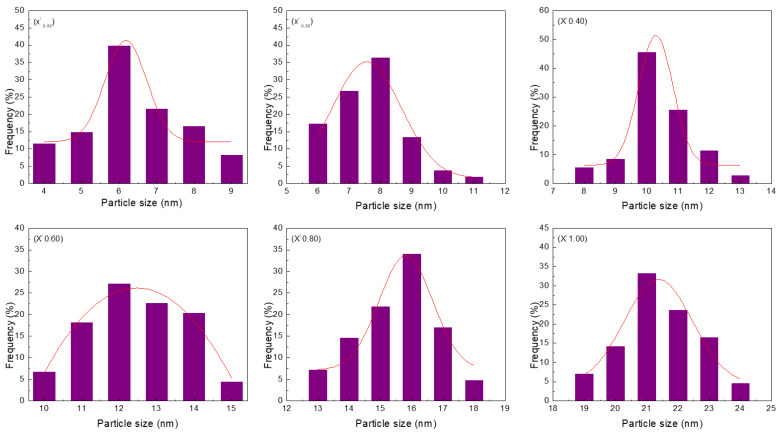
Particle size distribution of (x^/^_0.00_) Ce_0.00_Sn_1.00_O_2_, (x^/^_0.20_) Ce_0.20_Sn_0.80_O_2_, (x^/^_0.40_) Ce_0.40_Sn_0.60_O_2_, (x^/^_0.06_) Ce_0.60_Sn_0.40_O_2_, (x^/^_0.08_) Ce_0.80_Sn_0.20_O_2_, and (x^/^_1.00_) Ce_1.00_Sn_0.00_O_2_) nanoparticles calcined at 650 °C.

Transmission electron microscopy (TEM) was the basis for profiling the nanoparticle samples. This method is particularly useful because it ensures that the generated nanoparticles have a homogeneous round shape and size. Samples typically display uniformity in term of morphological features (x_0.00_–x_1.00_). Ce_x_Sn_1−x_O_2_ nanoparticles were subjected to calcination at 650 °C and their TEM images and particle size distributions are illustrated in Figure 3 and Figure 4, respectively. These images indicate that the existence of direct proportionality between the x values and particle size, with the increase in particle size being determined by contiguous particle aggregation, which in turn is caused by surface melting at elevated calcination temperature and x values.

The size uniformity and round shape of the Ce_x_Sn_1−x_O_2_ nanoparticles were confirmed by the results obtained. The standard process of nanoparticle sample production was established to be effective, with the sizes of the nanoparticles being influenced by the presence of PVP in a considerable amount based on the agglomeration and suppression mechanism [51]. The outcomes of the XRD and TEM analyses, obtained from the differently sized nanoparticles (from 6 nm to 21 nm), synthesized at 650 °C by increasing the x value, are outlined in Table 1. PVP is used as a stabilizer for particles and mediates nanoparticle nucleation and formation while also contributing to homogeneity [52,53,54,55]. As such, it is useful for restricting nanoparticle size, as well as for preventing nanoparticle aggregation [13,56,57,58,59]. Consequently, the particles increased in size when CeO_2_ was used in the sample (x = 0.20–1.00) due to the intensified agglomeration. Meanwhile, the reduction trend could be attributed to the fact that Ce^3+^ and Sn^2+^ have different ionic crystal radii.

### 3.3. SEM Analysis

**Figure 5:**
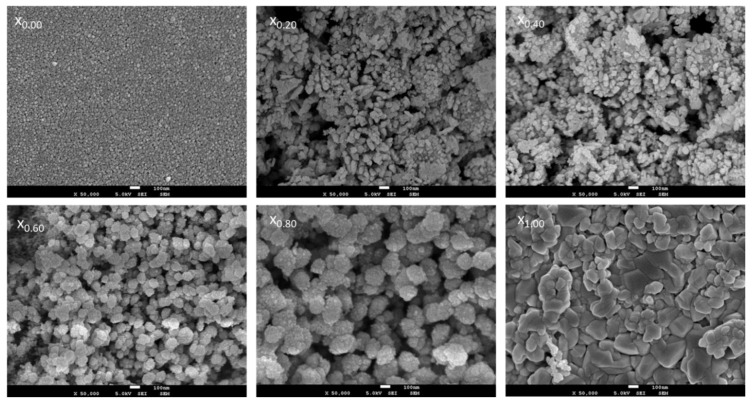
SEM images of (x_0.00_) Ce_0.00_Sn_1.00_O_2_, (x_0.20_) Ce_0.20_Sn_0.80_O_2_, (x_0.40_) Ce_0.40_Sn_0.60_O_2_, (x_0.06_) Ce_0.60_Sn_0.40_O_2_, (x_0.08_) Ce_0.80_Sn_0.20_O_2_ and (x_1.00_), Ce_1.00_Sn_0.00_O_2_) nanoparticles calcined at 650 °C.

The morphologies of Ce_x_Sn_1−x_O_2_ nanoparticles have been analyzed using scanning electron microscopy (SEM). Figure 5 illustrates micrographs associated with the Ce_x_Sn_1−x_O_2_ nanoparticles at every x value. The shapes of the prepared samples were almost spherical with regularities at x = 1.00, while the samples featured small grains and were spherical at x = 0.00. This finding was consistent with those reported in earlier studies [13,60]. In Figure 5, the images for x = 0.20 and x = 0.80 show samples of clusters formed with a round shape due to the fusion, breakdown, and overlapping of grains caused by the reduction in x value. Small particles agglomerated on large particles as shown in images where x = 0.60 and x = 0.80, due to the decreased amount of SnO_2_ that caused an agglomeration of SnO_2_ on CeO_2_.

### 3.4. XRD Analysis

**Figure 6:**
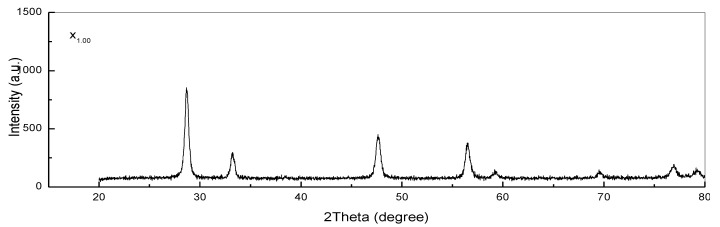

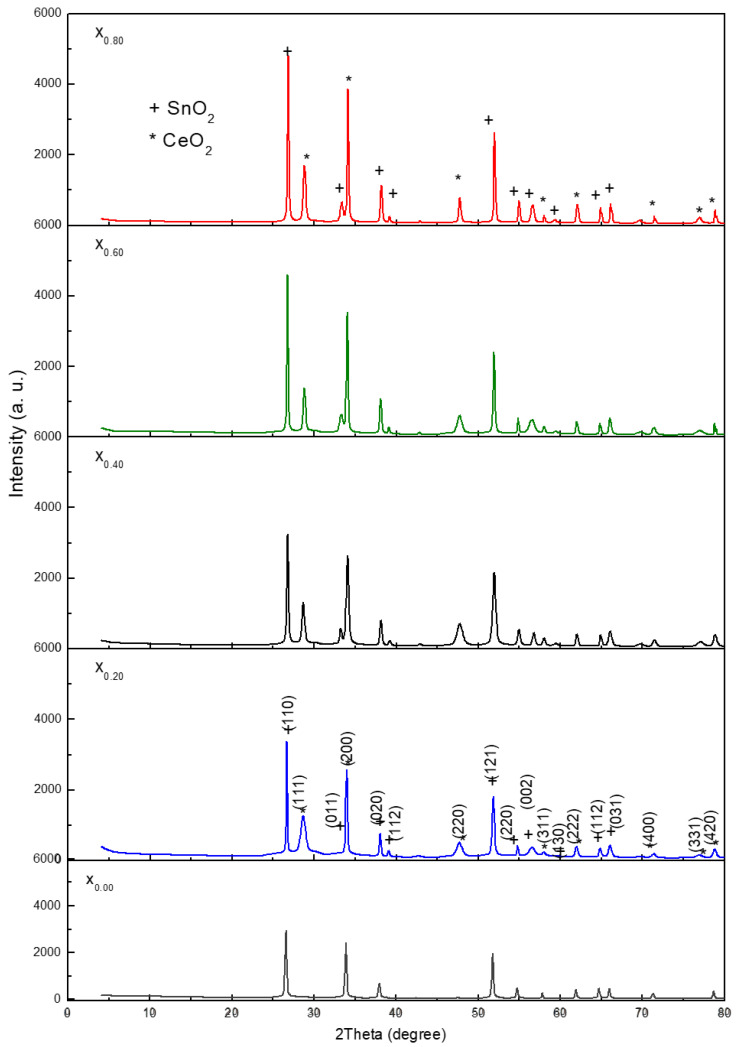
XRD patterns of (x_0.00_) Ce_0.00_Sn_1.00_O_2_, (x_0.20_) Ce_0.20_Sn_0.80_O_2_, (x_0.40_) Ce_0.40_Sn_0.60_O_2_, (x_0.06_) Ce_0.60_Sn_0.40_O_2_, (x_0.08_) Ce_0.80_Sn_0.20_O_2_ and (x_1.00_) Ce_1.00_Sn_0.00_O_2_) nanoparticles calcined at 650 °C.

The XRD patterns correlated with the Ce_x_Sn_1−x_O_2_ nanoparticles after 180 min of calcination at 650 °C are shown in Figure 6 (x_0.00_–x_1.00_), with the diffraction peak associated with SnO_2_ nanoparticles being denoted by the plus symbol, while the diffraction peak for the CeO_2_ nanoparticles is denoted by the star symbol. In the context of XRD patterns, the diffraction peaks represent standard values equivalent to the SnO_2_ and CeO_2_ compounds with tetragonal and cubic fluorite structures, respectively. The peaks associated with the SnO_2_ nanoparticles correspond to the (110), (011), (020), (121), (220), (002), (130), (112), (031), (022), and (231) planes, which is in agreement with JCPDS 00-041-1445 [4]. Meanwhile, the peaks associated with the CeO_2_ nanoparticles correspond to the (1 1 1), (2 0 0), (2 2 0), (3 1 1), (2 2 2), (4 0 0), (3 3 1), and (4 2 0) planes, which is consistent with the PDF Card No: 34-0394 data [14]. Furthermore, a mixture of SnO_2_ nanoparticle tetragonal structures and CeO_2_ nanoparticle cubic fluorite structures was exhibited by the generated Ce_x_Sn_1−x_O_2_ nanoparticles [61,62]. It must be noted that the XRD patterns of the samples did not reveal any contamination peak. Scherrer’s formula can be applied to determine the nanoparticle crystal size (*D*), as demonstrated as follows [6]:

*D* = (0.9λ)/(βcosθ), (1)

where the X-ray wavelength (1.5406 Å), the full width at half maximum, and the diffraction angle are respectively denoted by λ, β, and θ. Thus, an increase in the x value to 1.00 mmol cerium nitrate hexahydrate determined an enlargement in crystallite size from 5 to 19 nm. It can be deduced from the results that a rise in the x value yields a diffraction peak with a greater intensity, as presented in Figure 5 (x_0.20_–x_0.80_). Consequently, in relation to the nuclei, the enlarged particle size leads to an increase in crystalline volume ratio, thereby improving crystallinity [6,14].

### 3.5. FT-IR Analysis

**Figure 7:**
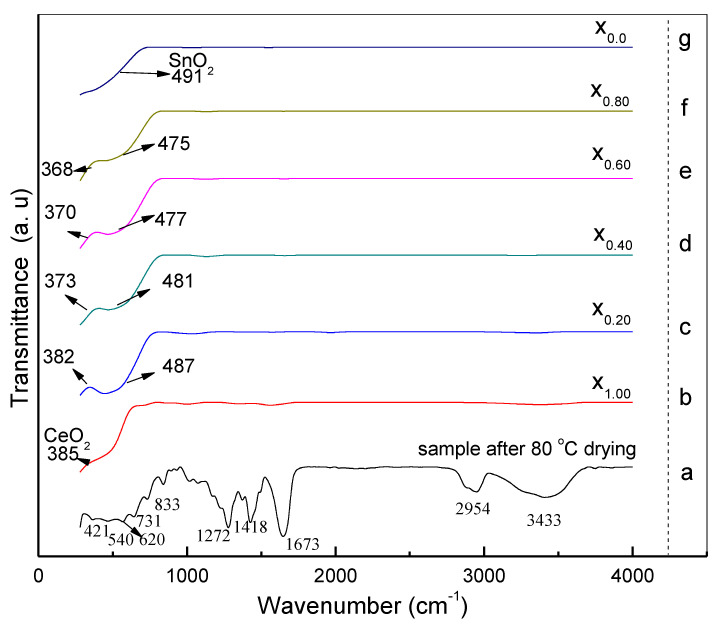
FTIR spectra of (**a**) the sample after drying at 80 °C ((**b**), x_1.00_) Ce_1.00_Sn_0.00_O_2_, ((**c**), x_0.20_) Ce_0.20_Sn_0.80_O_2_ ((**d**), x_0.40_) Ce_0.40_Sn_0.60_O_2_, ((**e**), x_0.60_) Ce_0.60_Sn_0.40_O_2_, ((**f**), x_0.80_) Ce_0.80_Sn_0.20_O_2_ and ((**g**), x_0.00_) Ce_0.00_Sn_1.00_O_2_ nanoparticles calcined at 650 °C.

The FT-IR spectrum related to the Ce_x_Sn_1−x_O_2_ samples has been studied in the range of 80–4000 cm^−1^. Figure 7a–g shows FT-IR spectra for the samples with different x values after drying at 80 °C and being calcined at 650 °C. In Figure 7a (sample after drying at 80 °C only) the absorption bands at wavenumbers 3433, 2954, and 1673 cm^−1^ were attributed to N–H, C–H, and C = O stretching vibrations [63]. The absorption band at 1418 cm^−1^ was attributed to C–H bending vibration initiated in the methylene group, and the peak at 1272 cm^−1^ was assigned to the C–N stretching vibration. The bands at 833, 731, and 620 cm^−1^ were mapped to NO^3-^ groups, with the vibrations generated by C–C ring and C–N=O bending [64,65]. The absorption bands at 421 and 540 cm^−1^ were assigned to Ce–OH band Sn–OH vibrations. Figure 7b–g show the disappearance of these peaks due to broadband absorption for the samples calcinated at 650 °C. The single absorption peak at 385 cm^−1^ in Figure 7b was attributed to Ce–O, and the other single absorption peak at 491 cm^−1^ in Figure 6g was attributed Sn–O. The double absorption peaks at 382, 373, 370, 368 cm^−1,^ and 487 cm^−1^ in Figure 7c–f were attributed to Ce–O, and Sn–O, where the wavenumber values for the sample spectra changed when the x values were increased. In regard to the x value increase, this was proved by the variable crystallinity exhibited by the generated Ce_x_Sn_1−x_O_2_ nanoparticles. All the bands characterizing the samples (i.e., x = 0.20–0.80) were included in the range of the Ce-O and Sn-O stretching vibrations, confirming the purity of the synthesized Ce_x_Sn_1−x_O_2_ nanoparticles. Thus, the samples were free of additional impurities [4,60].

### 3.6. Bandgap Analysis

**Figure 8:**
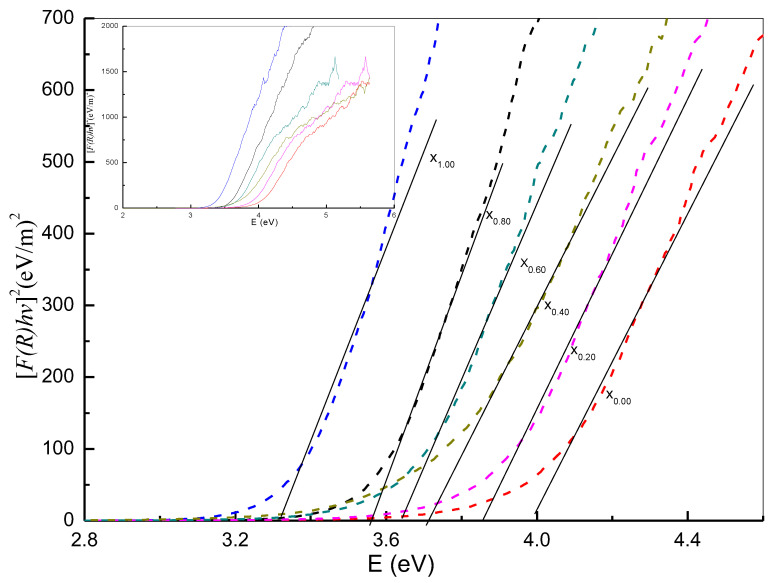
The energy bandgap of (x_0.00_) Ce_0.00_Sn_1.00_O_2_, (x_0.20_) Ce_0.20_Sn_0.80_O_2_, (x_0.40_) Ce_0.40_Sn_0.60_O_2_, (x_0.06_) Ce_0.60_Sn_0.40_O_2_, (x_0.08_) Ce_0.80_Sn_0.20_O_2_, and (x_1.00_) Ce_1.00_Sn_0.00_O_2_) nanoparticles calcined at 650 °C.

The procedure underpinning the Kubelka–Munk function consists of mapping the square of this function, namely, (F(R_∞_)hv)^2^, in relation to the energy and extension of the linear portion of the curve to F(R)^2^ = 0. As shown in Figure 8 (x_0.00_–x_1.00_), the Kubelka–Munk function facilitates the calculation of energy bandgaps for nanoparticles according to the diffuse reflectance spectra associated with samples exposed to a temperature of 650 °C. In this way, the band gap energy was generated for the oxide nanoparticles, with the energy bandgap values and x values being inversely correlated. Quantum size effects explained the rise in the bandgap energy value, whereas the transitions among the partially suitable valance and conduction bands for Ce^3+^ ion d-shell electrons explained the decrease in the band gap energy. Given the aspects addressed above, it is a complicated matter to eliminate the effect of particle size with regard to the band gap. Indeed, the material properties and band structure can be altered, where, owing to a particle size decrease, bandgap reduction is directly related to size enlargement. Disruption in the s-electron and p-electron conduction bands occurs at higher energy levels, leading to the possibility of superimposition when the particles are of a small size. In term of the Fermi-level distance, the larger the distance from the particle center, the lower the nuclear potential for electron conduction is. Hence, the absorption energy is equivalent to the conduction band energy in the case of transitions with permissible quantum numbers. In this work, band gap values were reduced whilst x values were increased to make it easier to conduct a comparative analysis, as shown in Table 1; however, flawed states may be enhanced at elevated x values, resulting in a higher absorption coefficient. Furthermore, the characteristics of the optical nanomaterial and related electronic structures can be modified by the fields of the electron hole pairs generated by photon absorption.

## 4. Conclusions

The results obtained in this paper have proven that calcination is an effective technique for the synthesis of Ce_x_Sn_1−x_O_2_ nanoparticles. In addition, X-ray diffraction analysis revealed that, at every x value, the Ce_x_Sn_1−x_O_2_ nanoparticles exhibited a cubic fluorite structure for CeO_2_ and a tetragonal structure for SnO_2_. A direct correlation was established between the nanoparticle size and the x value, with sizes ranging between 6 and 21 nm at x values between 0.00 and 1.00. The double absorption peaks were attributed to Ce–O and Sn–O and confirmed the purity of the Ce_x_Sn_1−x_O_2_ nanoparticles. A shift in the wavenumber for the sample’s nanoparticles spectra at increasing x values was documented. The novel thermal treatment method confirmed that the crystallinity of the Ce_x_Sn_1−x_O_2_ nanoparticles has been produced. Furthermore, the major vibrational modes displayed by Ce-O and Sn-O were identified via FT-IR analysis, while UV-vis absorption analysis revealed that, as the x value increased, the energy band gap diminished. Moreover, the smaller particle size was derived from a lower x value and the smaller energy band gap was derived from a higher x value. It can thus be concluded that products at lower x values can have antibacterial activity applications as the smaller particles can diffuse through the cell wall faster, while the products at higher x values can have solar cell energy applications due to more electrons can be generated at the bigger particle size.

## Figures and Tables

**Table 1:** XRD and TEM results for Ce_x_Sn_1−x_O_2_ nanoparticles of various x values when synthesized at 650 °C.

x Values	Sample Concentrations Ce_x_Sn_1−x_O_2_ At 650 °C and 4.5 gm of PVP	Particle Size by TEM (nm)	Crystallite Size by XRD (nm)	Energy Bandgap (eV)
0.00	Ce_0.00_Sn_1.00_ O_2_	6 ± 2	6	3.97
0.20	Ce_0.20_Sn_0.80_ O_2_	8 ± 3	7	3.86
0.40	Ce_0.40_Sn_0.60_ O_2_	10 ± 2	9	3.72
0.60	Ce_0.60_Sn_0.40_ O_2_	12 ± 4	10	3.64
0.80	Ce_0.80_Sn_0.20_ O_2_	16 ± 3	14	3.56
1.00	Ce_1.00_Sn_0.00_ O_2_	21 ± 2	19	3.40
